# Fiber-Optic Point-Based Sensor Using Specklegram Measurement

**DOI:** 10.3390/s17102429

**Published:** 2017-10-24

**Authors:** Jiao-Jiao Wang, Shao-Cheng Yan, Ya-Ping Ruan, Fei Xu, Yan-Qing Lu

**Affiliations:** College of Engineering and Applied Sciences, Nanjing University, Nanjing 210093, China; wangjiaojiao0719@163.com (J.-J.W.); njushchyan@163.com (S.-C.Y.); feixu@nju.edu.cn (F.X.); yqlu@nju.edu.cn (Y.-Q.L.)

**Keywords:** speckle interferometry, laser patterns, speckle correlation

## Abstract

Here, we report a fiber-optic point-based sensor to measure temperature and weight based on correlated specklegrams induced by spatial multimode interference. The device is realized simply by splicing a multimode fiber (MMF) to a single-mode fiber (SMF) with a core offset. A series of experiments demonstrates the approximately linear relation between the correlation coefficient and variation. Furthermore, we show the potential applications of the refractive index sensing of our device by disconnecting the splicing point of MMF and SMF. A modification of the algorithm in order to improve the sensitivity of the sensor is also discussed at the end of the paper.

## 1. Introduction

Fiber sensors have been widely investigated in many fields. Typically, there have been progressive reports on the excellent performance of fiber Bragg gratings [1], long-period fiber gratings [2], Fabry–Pérot interferometers [3] and so on. The analysis of these kinds of sensors mostly relies on monitoring the shift of wavelength captured by the optical spectrum analyzer (OSA). Compared with technologically advanced sensors, there exist newly developed sensors relying on speckle analysis techniques. Fiber-optic specklegram sensors are lower in cost, and use a lightweight camera, without the assistance of OSA. In this paper, we introduce a certain kind of fiber-optic specklegram sensor, as an alternative to the aforementioned apparatuses.

Fiber specklegram sensors are a category of optical fiber sensors that make use of multimode interference analysis in order to retrieve information about the waveguide status [4]. As the spatial and temporal characteristics of the speckle field are affected by the light guidance conditions, it is possible to correlate the specklegram changes with the environment change of the optical fiber [5]. The application of fiber specklegram sensors has been demonstrated to detect several physical variables, including micrometric displacements [6], vibration [7,8], temperature [9] and so on. The practical applications are also reported in human–robot interaction, such as tactile arrays [10] and devices for defining hand movements [11]. There are also a large number of conventional optical techniques, such as classical interferometry, speckle shearing interferometry [12] and electronic speckle pattern interferometry [13], which are used for temperature and pressure tests. Some previous works rely on the analysis of either the stripes in speckle patterns [5], or the fringe shift [9] while others introduce a specific parameter to evaluate speckle changes [6,7,10]. Previous research into speckle-based sensors generally focuses on detecting perturbation along multimode fibers (MMF). There was a report on a laser-based, noncontact and remote temperature sensor suitable for a wide range of temperature measurements in 2014 [14]. Another sensor designed for applied load measurement was reported in 2016 [15] which was also based on the speckle analysis technique.

In this paper, we introduce a newly developed fiber-optic point-based temperature and weight sensor by measuring the speckle changes under different environmental conditions. Compared with the aforementioned specklegram sensors, our specklegram sensor consists of a SMF and a MMF and the sensing point is the connecting area. Some wavelength-shift-type sensors that take advantage of similar SMF–MMF configurations have been demonstrated in the past years [16,17,18]. Our specklegram-type sensor has two main advantages. On the one hand, apart from detecting the change along the MMF, the point-based sensor can reduce the instability induced by the inherent fluctuation. Therefore, integration of the point-based sensor is more practical and can be applied to sense the refractive index change. On the other hand, compared with spatial light used in previous work, the SMF–MMF model turns out to be more stable and easier to integrate. In the proposed sensor, higher-order modes are excited by splicing a multimode fiber to a single-mode fiber with a fiber core offset. We also demonstrate the refractive index sensing of our device by disconnecting the splicing point and leaving the coupling area exposed to a liquid environment. 

All of the experiments demonstrate the approximately linear relation between correlation coefficient and variation, and the sensing response to temperature or weight reveals a more linear relationship compared to previous works presented in Refs. [14,15].

## 2. Fiber Specklegram Analysis

When a laser beam with sufficient coherence length is launched into a MMF, it generates a random interference pattern or speckle field at the fiber end face. When light travels from a SMF to a MMF through a spliced point or just through coupling across the gap (in other words, a “conversion point”), it excites higher modes in the MMF. Aligning the fibers with a core offset can also excite more higher-order modes and as a result, interference between modes generates a random pattern. When we change the environment around the “conversion point”, the ratio and energy distribution of modes varies correspondingly. The speckle field is affected by the phases and energy distribution of modes, leading to fluctuations in both location and intensity of the output specklegrams.

**Figure 1:**
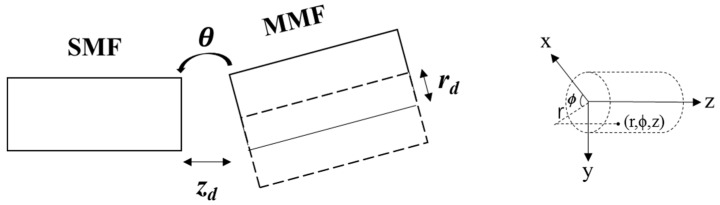
Schematic diagram of the single-mode fiber (SMF) coupled to the multimode fiber (MMF) with a gap *z_d_*, along with a lateral core offset *r_d_* and a slight tilt *θ*.

In the graded index, the modal field depends on all three cylindrical polar coordinates *r*, ϕ and *z*, as shown in Figure 1, and the modal field has the separable form:

(1) ψ(r,ϕ,z)=ψ(r)ψ(ϕ)exp(−iβz)

Conventionally, the Gaussian field emitted from the left single-mode fiber is described by Equations (2) and (3) [19]. The gap between two fibers *z_d_* can be either zero or nonzero. The parameter *r_d_* describes the core offset along the radius and the *θ* is the inclined small angle to the fiber axis, as shown in Figure 1. The efficiency of each *ψ_m,v_* mode excited by the fraction of Gaussian beam power in the right multimode fiber *η_m,v_* is described in Equation (4) [19]. The parameters *r_d_* vary with changes in temperature, stress or refractive index, resulting in different constitutions of the input beam, while *θ* remains unchanged throughout the process. For different modes *ψ_m,v_*, the energy distribution is determined by Equation (4) and changes with input *ψ_Gauss_* described in Equation (2). As the situation varies, specklegrams shown at the fiber end face appear to be different.

(2) $$\psi_{G\mathrm{auss}}{(r,r}_{d},\phi )=\frac{\sqrt{2/\pi }}{\omega_{0,0}}\exp\left(-\left\{{\left[r\cos(\phi )+r_{d}\right]}^{2}+{\left[r\sin(\phi )\right]}^{2}\right\}*\frac{1}{\omega_{0,0}^{2}}\right),\;\theta =0$$

(3) $$\psi_{G\mathrm{auss}}(r,\theta ,\phi )\approx \frac{\sqrt{2/\pi }}{\omega_{0,0}}\exp\left[-\frac{r^{2}}{\omega_{0,0}^{2}}+jkr\theta \cos(\phi )\right],\;r_{d}=0$$

(4) $$\eta_{m,v}=\frac{{\left|\int_{0}^{2\pi }\int_{0}^{\infty }\psi_{G\mathrm{auss}}\psi_{m,v}\cos(m\phi )rdrd\phi \right|}^{2}}{{\int_{0}^{2\pi }\int_{0}^{\infty }{\left|\psi_{G\mathrm{auss}}\right|}^{2}rdrd\phi *\int_{0}^{2\pi }\int_{0}^{\infty }\left|\psi_{m,v}\right|}^{2}\cos{\left(m\phi \right)}^{2}rdrd\phi }$$

where integer *m* is a mode order in the azimuthal direction, and *v* is a mode order in the radial direction, and *ω*_0,0_ is the spot size radius of the first mode.

The principle of operation is based on analyzing the laser speckle through a digital correlation technique [20,21]. Under the guidance of the Equation (5) [22], the surrounding environment is determined by correlating the intensities of the speckle pattern recorded under the unknown condition with a reference speckle pattern recorded previously.

(5) $$C=\frac{\sum_{k=1}^{N}\sum_{l=1}^{M}\left[I_{R}\left(k,l,t_{0}\right)-{\bar{I}}_{R}\left(t_{0}\right)\right]\left[I_{0}\left(k,l,t\right)-{\bar{I}}_{0}\left(t\right)\right]}{\sqrt{\left\{\sum_{k=1}^{N}\sum_{l=1}^{M}{\left[I_{R}\left(k,l,t_{0}\right)-{\bar{I}}_{R}\left(t_{0}\right)\right]}^{2}\right\}\left\{\sum_{k=1}^{N}\sum_{l=1}^{M}{\left[I_{0}\left(k,l,t\right)-{\bar{I}}_{0}\left(t\right)\right]}^{2}\right\}}}$$

where *I_R_*(*k*, *l*, *t*_0_) and *I*_0_(*k*, *l*, *t*) are, respectively, the intensities of the speckle patterns recorded by the digital device before (at initial condition *t*_0_) and after applying the fluctuation to the fiber (at applied condition *t*). ${\bar{I}}_{R}\left(t_{0}\right)$ and ${\bar{I}}_{0}\left(t\right)$ are their mean values. The change in correlation coefficient is computed from the obtained correlation value using the equation below [23]:

Δ*C* = 1 − *C*. (6)

Mathematically, the correlation coefficient *C* is a number that quantifies a type of correlation and dependence, indicating statistical relationships between two or more values in fundamental statistics. The coefficient that we use in this paper is “Pearson Correlation Coefficient”, a measurement of the strength and direction of the linear relationship between two variables. Typically, if the two compared specklegrams change significantly, the coefficient *C* appears to be smaller. In other words, if the environment changes more, *C* gets smaller, revealing a negative relationship. Here, to be consistent with common standards, we take Δ*C* as a criterion to obtain a positive correlation [23].

In our experiments, for each detected temperature, 10 speckle patterns are sampled at 10 Hz. For each of these speckle patterns, the correlation coefficient with every one of the reference frames is computed and is time-averaged [23].

## 3. Experimental Procedure and Results

**Figure 2:**
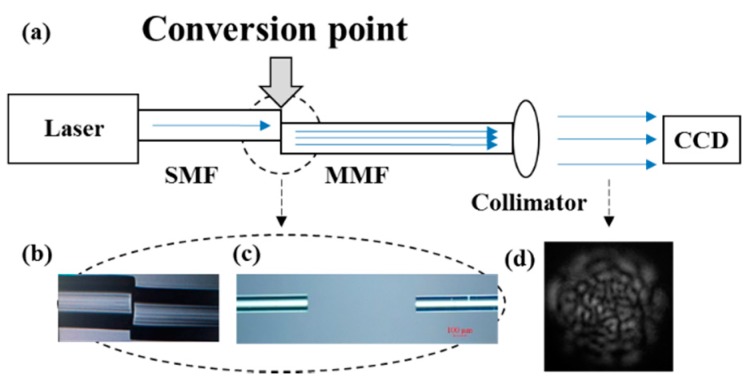
(**a**) Schematic diagram of the experimental setup of the fiber specklegram sensor; (**b**) Micrograph of the spliced specimen with a core offset *r_d_*, where *z_d_* is zero; (**c**) Micrograph of the aligned specimen with a core offset *r_d_*, where *z_d_* is nonzero; (**d**) The output specklegram detected by a charge-coupled device (CCD).

The experimental configuration shown in Figure 2a is designed to measure temperature and pressure changes. The tunable laser source (1412S003, Santec, Aichi-ken, Japan) is introduced to the system through a single-mode fiber (SMF-28, Corning, NY, USA) at 1550 nm. Then, a 50 cm MMF (105 μm core, 0.22 NA, Nufern, East Granby, CT, USA) is spliced to the SMF with a core offset (~26 μm), to excite hundreds of higher-order modes which can strengthen the effect of interference. Details of the spliced point (the “conversion point”) are shown in Figure 2b, where the status of guided modes is changed. For the temperature measurement, a thermal controller is used to produce different temperature distributions at the “conversion point”. As for pressure measurement, hundreds of grams are applied on the splicing point. The aligned specimen is shown in Figure 2c, which can be used to detect the refractive index change. The output speckle field at the end of the MMF is sampled using a charge-coupled device (CCD, MicronViewer 7290A, Electrophysics, Sofradir, Fairfield, NJ, USA), and the speckle image is shown in Figure 2d (cropped into a square).

**Figure 3:**
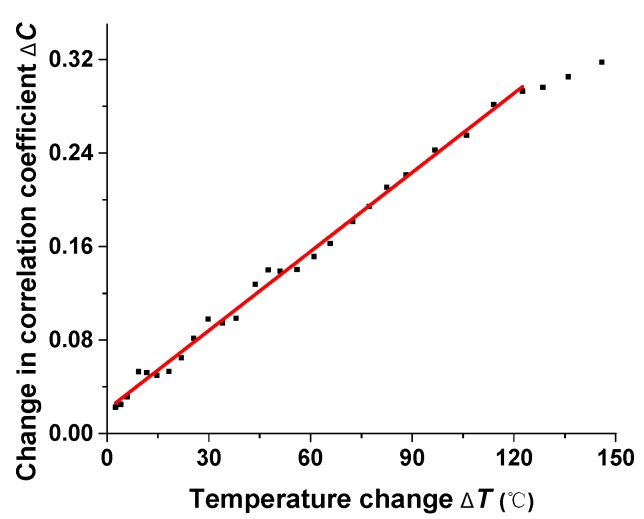
Sensing response for temperature change.

Figure 3 illustrates the sensor response for temperature change. When the “conversion point” is heated, the intensity distributions of modes transmitted to the MMF vary. The spatial and temporal characteristics of the speckle field are affected by the status of modes, and the speckle image seems much more different with the original reference speckles, resulting in a larger Δ*C*. The reference speckle is recorded at room temperature (about 25 °C), and as expected, the curve in Figure 3 represents the approximately linear positive relationship between temperature change and Δ*C*. The sensitivity is about 0.00225/°C. When the environmental temperature is higher than 120 °C, the Δ*C* shows a nonlinear response, which may be attributed to the intrinsic limitation of the coefficient *C*. Additionally, temperature change also interferes when applied along the MMF. The heat along the MMF changes the phase shift of propagating modes, and results in a more sensitive response according to additional experiments. However, the instability of the MMF brings difficulty in practical use. Under such considerations, we can suppress the influence of the MMF by packaging it with thermal insulation material. Moreover, our sensor is preferred to detect the temperature of a small object. 

**Figure 4:**
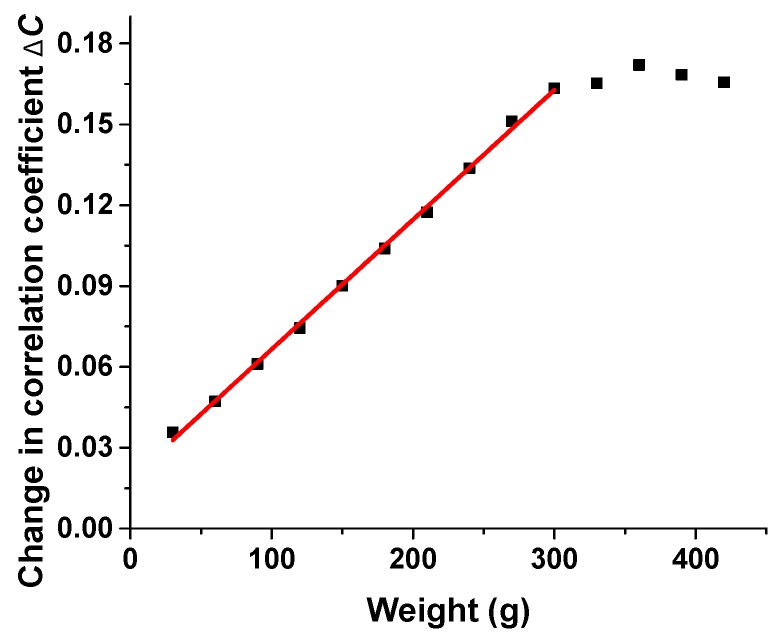
Sensing response for pressure change.

The pressure response is similar to the temperature response, which also relies on the effect of a coupling coefficient. When the load is placed on the “conversion point”, the output speckles change with a positive linear relationship between pressure change and Δ*C*. In order to reduce the influence of the surrounding environment, the sample point is sealed in nail polish, and it is hard to figure out the actual pressure loaded on the point precisely. We can place a mold on the “conversion point” with a small contact point. The mold consists of a supporting point and a platform, which can guarantee a more stable applied load and keep the pressure directly applied on the sensing point. To describe the relationship between Δ*C* and variation of pressure, we take the loaded weight as pressure applied to the “conversion point”. As a result, here we only focus on the relationship between loaded weight and Δ*C*. The linear relationship between pressure and Δ*C* is shown in Figure 4, where the response range is 300 g, and sensitivity is 0.0004812/g. 

**Figure 5:**
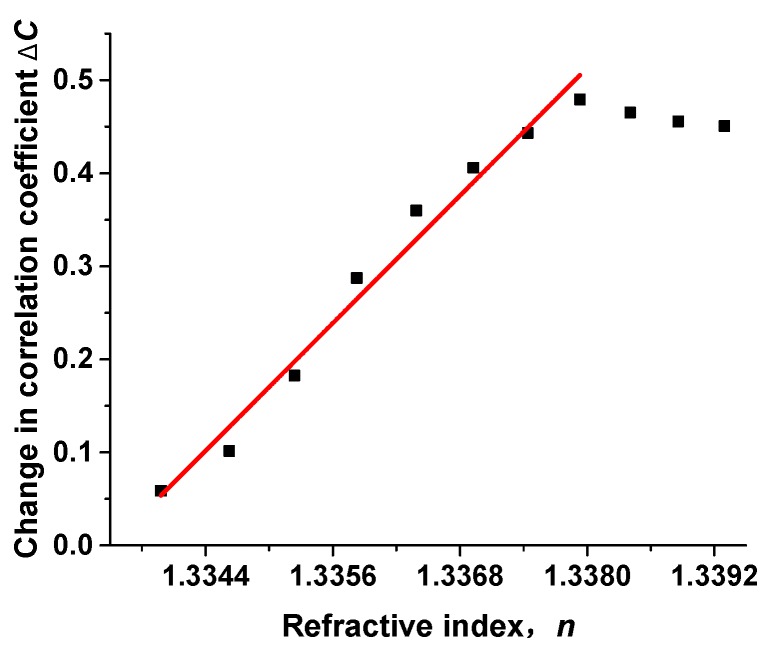
Sensing response for different refractive indices in the gap.

The apparatus proposed in Figure 2c also works when aligning the fibers directly with a gap *z_d_* and a slight core offset *r_d_*, generating higher-order modes. Compared with the speckle-based sensors reported before, the point-based sensor proposed in this paper can be used for the detection of the refractive index. The proposed setup is shown in Figure 2a and details of the aligned point are shown in Figure 2c. The gap between SMF and MMF in this experiment is about 884 μm. With a gap between fibers, it can be immersed in different kinds of solutions. Eleven ethanol solutions with different concentrations (2.44%, 4.76%, 6.98%, 9.09%, 11.11%, 13.04%, 14.89%, 16.67%, 18.36%, 20% and 21.57%) are used in this experiment. The corresponding refractive indices (calculated using water at 25 °C as reference for the 1550 nm wavelength) are 1.33398, 1.33462, 1.33524, 1.33583, 1.33639, 1.33693, 1.33744, 1.33793, 1.33841, 1.33886, 1.3393, respectively. The reference pattern is recorded in pure water, as Figure 5 shows, Δ*C* increases with the change of refractive index of the sample. The response range is 0.0046, and sensitivity is 114.2215/RIU.

For the setup to detect the refractive index change, the gap between the fibers works as an important parameter. On the one hand, a larger angle of divergence is obtained and results in higher-order modes excited in the MMF with a longer gap [19]. On the other hand, a longer gap provides a larger range of reaction for the solution and immersed fibers. As a result, we can get a more sensitive sensor with a larger gap. Nevertheless, the loss of coupling efficiency is a drawback.

The success of the refractive index sensor is attributed to the point-based design. The sensitivity to refractive index change can be applied to different fields, such as in measuring the progress of a chemical reaction where different ratios of reactant cause a change of refractive index, or alternatively, in biological measurements. 

Here, we propose a kind of point-based sensor for the measurement of temperature, pressure and refractive index. It should be noted that this method has the limitation of crosstalk, and can be only used for a specific field to guarantee a more precise detection. In fact, it is a common challenge for most kinds of fiber sensors. To solve the problem, one favorite method is to add some compensating fibers in order to calibrate other parameters.

## 4. Discussion

The electric field at any point on the exit face of the fiber consists of a sum of a multitude of individual field contributions:

(7) $$E\left(r,\phi ,L\right)=\sum_{m}A_{m}\psi_{m}\left(r,\phi \right)\exp\left(-i\beta_{m}L-\omega t\right)$$

where *A_m_* is the amplitude of the *m*th guided mode with spatial profile *ψ_m_* and propagation constant *β_m_*, and *L* is the length of the fiber [24]. The speckle used in our experiments is expected to spread randomly. If there are more propagating modes, the interference with each other can be more random on the level of statistics. A MMF with a common length is adequate to provide the interaction length for interference. Consequently, a shorter input laser wavelength or a larger-core MMF would have the possibility to excite more modes, and to generate better speckle patterns with more interference information. Moreover, we also find that there is a reduction in the contrast ratio with a longer fiber because of the increased disturbance of the environment interacting with the MMF. As a result, we find it will be appropriate to use a 0.5~1 m MMF, to guarantee the imaging quality. In practical use, we have to calibrate its length according to the given situation so that it can keep a relatively stable performance.

Due to the intrinsic limitation of the coefficient *C*, there always exists a limitation where the coefficient *C* cannot work. That limitation seems to present a trade-off between the range and the sensitivity of the aforementioned sensor. However, there does exist a way to improve the range and sensitivity by optimizing the algorithm.

Specklegram measurement is based on correlating the de-correlation of the speckle pattern at a specific condition with that of a reference speckle pattern which was recorded at confirmed conditions. The larger the size of the speckle pattern, the more information will be recorded. Therefore, to increase the range, we can use a larger magnification, consequently increasing the speckle size at the detector. In large patterns, a slower change in speckle correlation leads to a larger response range [14]. We can also extend the sensing dynamic range by speckle pattern division and then analyze the sub-images [4].

Furthermore, there are some ways to improve sensitivity in order to detect minor changes more efficiently. 

**Figure 6:**
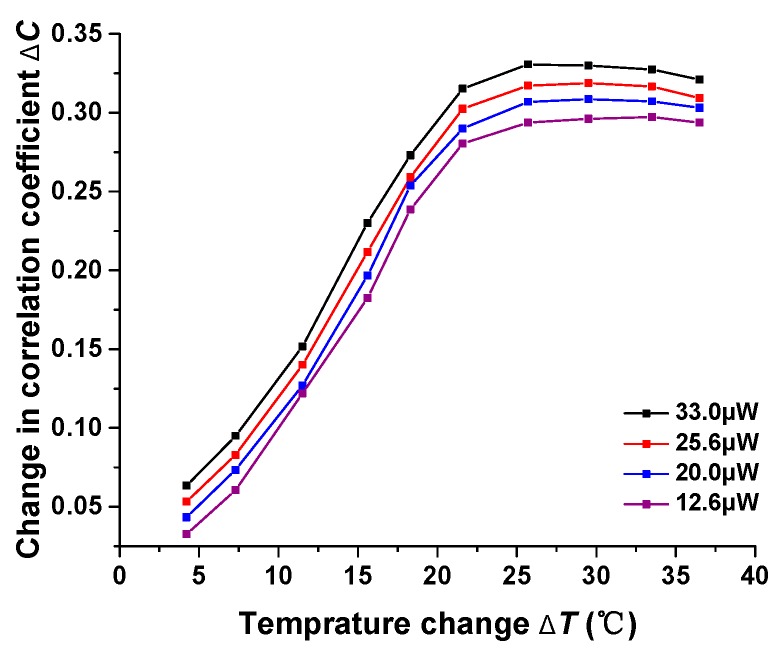
Temperature response under different laser powers.

A different laser power generates patterns with different illumination, resulting in a different response performance. As is illustrated in Figure 6, when, under the same experimental conditions, we adjust the input laser power to 33.0 μW, 25.6 μW, 20.0 μW and 12.6 μW respectively, the corresponding responses vary from each other in terms of sensitivity. In the dim patterns, the fluctuation of each pixel is averaged to be large compared with the background, so reducing the power of the laser can improve the ordinate and thus improve the sensitivity. However, it is obvious that the input power cannot be raised too high, otherwise, it will damage the CCD and bring about a bad contrast ratio.

**Figure 7:**
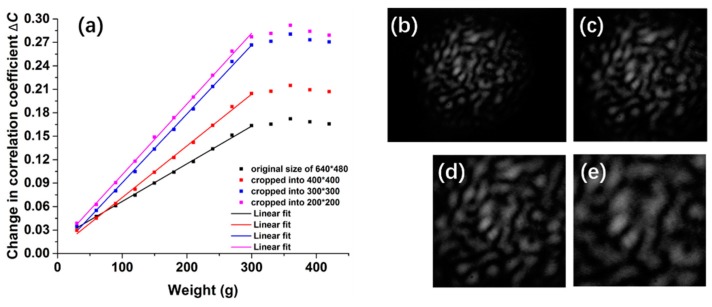
(**a**) Pressure response of different sizes of patterns; (**b**) Original pattern recorded directly by CCD; (**c**) Pattern cropped into 400 × 400; (**d**) Pattern cropped into 300 × 300; (**e**) Pattern cropped into 200 × 200.

For the same sampled speckle patterns, the optimizing algorithm can also help us figure out “more useful” information. For the analysis of pressure measurements in the data shown in Figure 4, we can improve its sensitivity by shearing the sampled patterns or by rejecting the pixels with little change. The original pattern recorded by CCD is shown in Figure 7b, which is 640 × 480 pixels. It is apparent that some pixels remain black, without being lit by the input laser, which are deficient in information. When we crop the original pattern into smaller ones (from the center of the speckle), such as the sizes 400 × 400, 300 × 300, 200 × 200, the response curve in Figure 7a shows a larger slope and reveals better sensitivity to the change of pressure.

**Figure 8:**
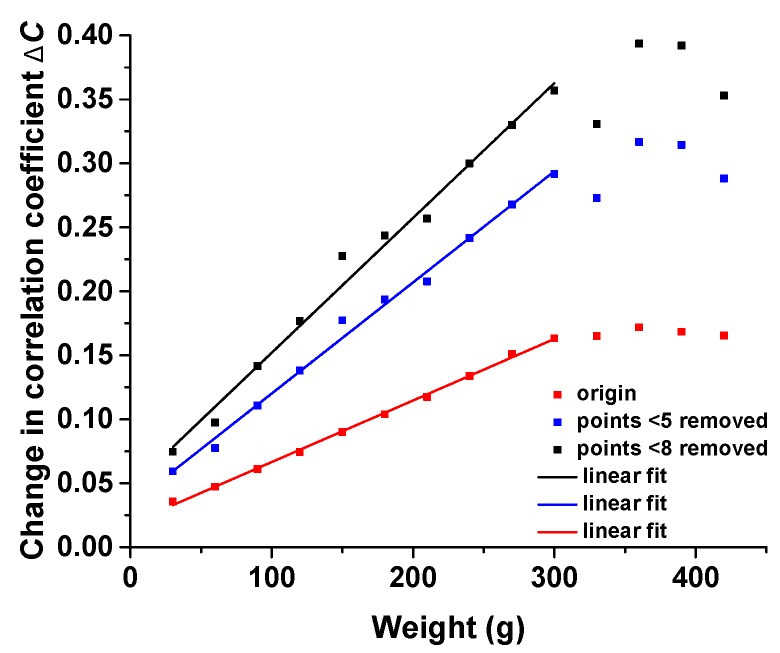
Pressure response under different degrees of reduction.

Higher sensitivity can also be obtained if we select informative patterns by removing the specific pixel which seems “unchanged”. In the noise reduction program, we can set a threshold to identify the bad points. The speckle patterns are mottled with black subregions, where the illumination of these pixels remains unchanged. To eliminate these points with little fluctuation, we can remove the bad data points which change within 5 or 8, and compare the optimized results with the original response. The curves in Figure 8 reveal the improvement of sensitivity by removing useless data. However, it indicates two drawbacks. The errors are magnified, and the linear fit seems to fit less well, demonstrating that there are possible limitations to the noise reduction program.

## 5. Conclusions

In summary, we propose a fiber-optic point-based method to measure temperature, pressure, and the refractive index, through the analysis of output specklegrams. We have also conducted some research on the improvement of sensor sensitivity and its dynamic range for practical applications. Compared with technologically advanced sensors that rely on the analysis of wavelength shift from an optical spectrum analyzer, the proposed speckle-based sensor is more compact, lightweight, and low-cost, revealing a good linear response to the change of environment. Owing to the stability of fibers and the “conversion point”, this sensor is also suitable for determining the remote situation where a compact and accurate integrated sensor is required. However, this sensor represents a proof-of-concept aimed at highlighting the detection mechanism. The influence of environment change, instability of MMF, and calibration for different detecting situations should be overcome through apparatus improvement and algorithm optimization for practical use in the future.
